# Ultrasound Imaging for Diaphragm Function in a Population of Healthy Infants: A Short Observational Report

**DOI:** 10.3390/diagnostics13061095

**Published:** 2023-03-14

**Authors:** Danilo Buonsenso, Francesco Mariani, Rosa Morello, Gianmaria Cammarota, Cristina De Rose, Piero Valentini, Anna Camporesi, Luigi Vetrugno

**Affiliations:** 1 Department of Woman and Child Health and Public Health, Fondazione Policlinico Universitario A. Gemelli IRCCS, Largo A. Gemelli 8, 00168 Rome, Italy; 2 Centro di Salute Globale, Università Cattolica del Sacro Cuore, 00168 Rome, Italy; 3 Department of Anaesthesia and Intensive Care Medicine, University of Perugia, 06100 Perugia, Italy; 4 Department of Pediatric Anesthesia and Intensive Care, Children’s Hospital “Vittore Buzzi”, 20154 Milan, Italy; 5 Department of Anesthesia, “SS Annunziata” Hospital, Via dei Vestini, 66100 Chieti, Italy; 6 Department of Medical, Oral and Biotechnological Sciences, University of Chieti-Pescara, 66100 Chieti, Italy

**Keywords:** diaphragm ultrasound, children, diaphragm thickening fraction

## Abstract

Introduction: Diaphragm ultrasound is increasingly used in adults, and more recently in pediatric practice. However, normal diaphragm parameters in healthy infants are unknown. This was a prospective observational pilot study aiming to define the normal diaphragm ultrasound characteristics in healthy infants during the first 6 months of life. Methods: We recruited healthy neonates at 7 to 15 days of life, who were followed until the sixth month of life, undergoing five assessments in different time points. The measurements included diaphragm thickness at end expiration (TEE) and at end inspiration (TEI). The thickening fraction (TF) was calculated as (TEI-TEE)/TEE and expressed as a percentage, and as (TEI-TEE)/TEI. Results: A total of 37 toddlers, 16 of which were females (43.2%), were enrolled. Thirty-four children (91.9%) were of Caucasian ethnicity and the median gestational age was 38.4 (35.7–40) weeks. Normal TEE, TEI, and TF have been provided for each time point. Conclusion: We provided new insight regarding data about thickness and thickening function in healthy children to be used for future physiologic and pathologic pediatric studies.

## 1. Introduction

The diaphragm is the most important respiratory muscle in the human body. It exerts its activity during inspiration, generating intrathoracic negative pressure that attracts air into the lung [1]. If, on the one hand, during fetal life, diaphragmatic movement is unnecessary to sustain gas exchange, on the other hand, fetal breathing activity is essential to develop and “train” this muscle [2]. From a physiological point of view, the diaphragm acts as a piston during inspiration and generates trans-diaphragmatic pressure (Pdi, cm/H_2_O) [3]. A double-balloon probe can monitor the difference between trans-gastric and trans-esophageal pressure and measure their effort in terms of trans-diaphragmatic pressure (Pdi), offering an intriguing mode to look at this vital respiratory system muscle, but invasively [4]. Only specialized centers with research purposes had the equipment to estimate this measure of muscle strength in adults and, rarer, in children. Ueki et al. first described that, in adults, diaphragm thickness measured with ultrasound correlated well with inspiratory mouth pressure [5]. They found that the mean (SD) thickness was 4.5 (0.9) mm at total lung capacity, 1.7 (0.2) mm at functional residual capacity, and 1.6 (0.2) mm at residual volume. There was a high degree of correlation between the thickening ratio and the pressure achieved during the maximum inspiratory maneuver (r = −0.82). Therefore, ultrasound has become an intriguing and easy way to evaluate the diaphragm, not invasively and at the bedside. In newborns and infants, the diaphragm needs to be thoroughly developed at birth, and its anatomy and physiology differ from later ages [6]. Data about diaphragm ultrasound at this age are scarce, and few studies have described the appearance and activity in newborns [7]. Furthermore, no study has prospectively evaluated this muscle’s structural and functional changes in the first six months of life. The present study aims to describe the development, in both structure and activity, of the newborn diaphragm through the first six months of life so as to provide a reference when using this smart tool in pathological, more complex situations.

## 2. Materials and Methods

This prospective, observational single-center study was conducted at a tertiary pediatric hospital level in Rome from 1 April 2019 to 30 April 2019, and those who were accepted to participate were followed until the sixth month of life. We recruited healthy neonates during the first post-discharge routine clinical visit that was performed in our institution at 7 to 15 days of life. Exclusion criteria included gestational age < 36 weeks and known congenital chest wall or diaphragmatic anomalies (e.g., congenital diaphragmatic hernia). IRB approval and written parental consent were obtained for every case before starting the study, and the European General Data Protection Regulation 2016/679 was (GDPR) respected. The study was unfunded.

### Diaphragm Ultrasound

**Figure 1:**
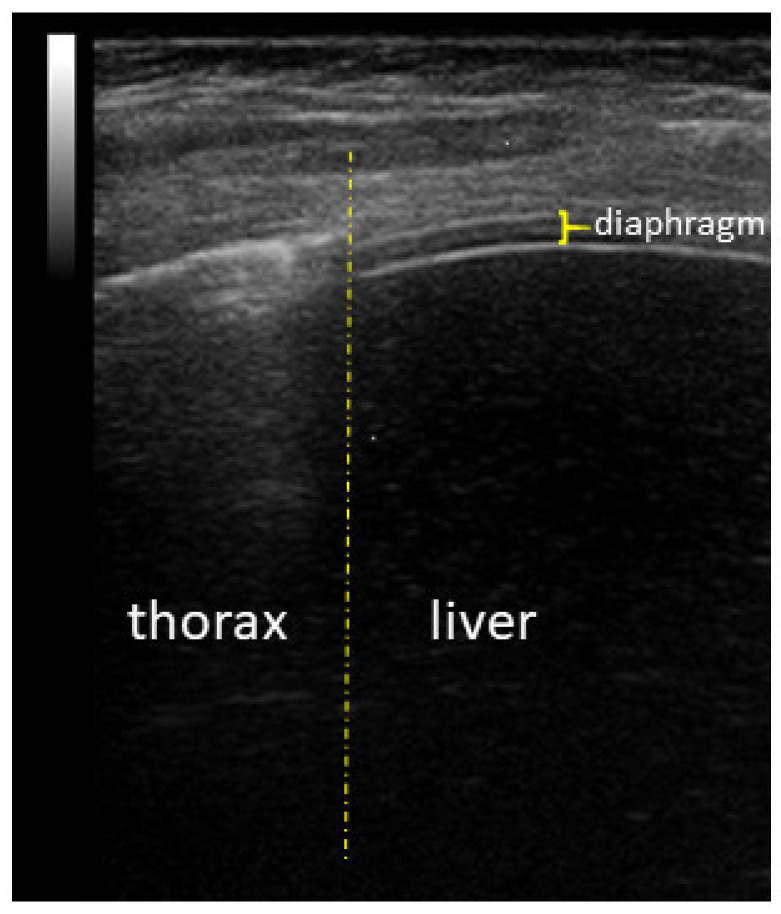
Ultrasound appearance of the diaphragm on the anterior axillar line.

Ultrasound diaphragm was performed using Esaote MyLab 40, as reported in our previous studies [6,8]. Briefly, ultrasonography examinations of the diaphragm were performed, placing the transducer in the ninth or tenth intercostal space near the midaxillary line and angled perpendicular to the chest wall. The posterior zone of apposition was assessed at 0.5–2 cm below the costophrenic sinus. The anterior zone of apposition was immature at this age. The diaphragm thickness was recorded in time motion mode. The diaphragm was outlined by the two clear bright parallel lines of the pleural and peritoneal membranes (Figure 1). 

The measurements included diaphragm thickness at end-expiration (TEE) and at end-inspiration (TEI). During M-mode imaging, the normally functioning diaphragm was represented as an echogenic line that moves freely during inspiration and expiration. Inspiration was identified on the sonographic tracing as upward flexion; expiration was identified as downward flexion. Estimation of diaphragmatic excursion was conducted by measuring the vertical distance between the upper border of the liver at the end of expiration to the upper border of the liver at the end of inspiration. This vertical distance represented right/diaphragmatic excursion [6]. Measurements were averaged out of three or more consecutive breaths on the last valid image recorded at the end of each period. Following these guidelines, the diaphragmatic thickness during inspiration and expiration was measured. The thickening fraction (TF) was calculated as (TEI-TEE)/TEE and expressed as a percentage, and as (TEI-TEE)/TEI.

## 3. Results

**Figure 2:**
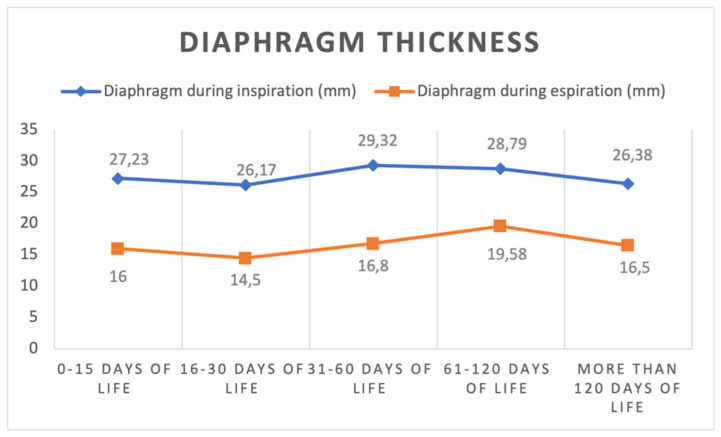
Diaphragm thickness during inspiration and expiration at different time points (mean values).

**Figure 3:**
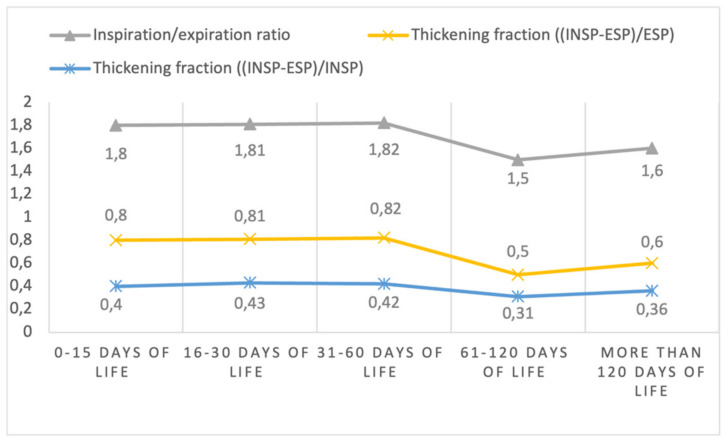
Diaphragm inspiration/expiration ratio and thickening fraction during inspiration and expiration at different time points (mean values).

Of 37 toddlers, 16 females (43.2%) were enrolled. Thirty-four children (91.9%) were of Caucasian ethnicity, and the median gestational age was 38.4 (35.7–40) weeks. Twenty of them (54.1%) were born by eutocic delivery, sixteen (43.2%) by cesarean section, and one (2.7%) by operative delivery. Two children (5.4%) were born by twin delivery, and 36 children (97.3%) were appropriate for gestational age (AGA) for weight, while only one (2.7%) was small for gestational age (SGA). Twenty-two children (59.5%) had an echography performed in the first 15 days of life, six (16.2%) in the period between the sixteenth and the thirtieth day of life, twenty-five (67.6%) in the period between the thirty-first and the sixtieth day of life, twenty-four (64.9%) in the period between the sixty-first and the one hundred and twentieth day of life, and eight children (21.6%) after the one hundred and twentieth day of life. The characteristics of the echography are reported in Table 1 and presented in Figure 2 and Figure 3.

## 4. Discussion

We studied the contraction of the diaphragm mainly in the posterior zone as the apposition zone was in development, as well as the accessory respiratory muscles. At this age, the diaphragm was flat, not a dome, as in the adult, and the ribs were horizontal. So far, a few studies have evaluated average values of diaphragm excursion or thickness in healthy children, unifying children from extensive age ranges (e.g., 1 month to 2 years or 6 to 12 years) [9]. This limited our ability to compare our data with previous data. However, the few studies including children aged 1 month to 2 years had a similar thickness as found in our cohorts, of about 3 mm [8,9,10].

Importantly, considering the rapid change in thorax circumference we saw in our study, and how this can affected compliance, studies should focus on smaller age groups to provide average reference values for pediatric practice. In this regard, although preliminary and based on a small number of children, our study is the first one providing a detailed assessment of diaphragm function in young infants. During this age, the diaphragm acts as a brake during expiration to prevent lung collapse. This is because the compliance of the chest is low and does not act with force over the lung in expiration to maintain end-expiratory lung volume (EELV). Importantly, we never documented diaphragm dysfunction as found in children with spinal muscular dystrophy [11,12]. These data are extremely needed in view of the growing understanding of the role of diaphragm ultrasound in adults in diagnosing diaphragm dysfunction. In adults, diaphragm dysfunction, documented by ultrasound, has been shown to predict success/failure from mechanical ventilation as well as an expert consensus about diaphragm and pediatric measurement is urgent needed [13]. Less studies have evaluated its role in pediatrics, although there is increasing understanding of the potential of this relatively new technique in pediatrics. For example, a few studies have documented how diaphragm function evaluated on ultrasound can be associated with different outcomes in children with bronchiolitis [6] or neuromuscular disorders [11,12], but even in mechanically ventilated children or cohorts undergone cardiac surgeries [4]. In addition, our understanding of the importance of the diaphragm, even during health, has increased during the last decade. A recent study demonstrated that during spontaneous breathing, the diaphragm could contract during expiration to preserve distal airway patency, while diaphragm paralysis favors end-expiratory lung collapse [14]. These findings may have practical implications such as the importance of assisting the diaphragm to prevent lung injury. However, our understanding of the physiologic function of the diaphragm during other conditions and developmental stages is needed. Therefore, studying these phases through ultrasound may open a new era for the management of children during different respiratory conditions or respiratory support. The small number of enrolled patients and the short follow-up (up to six months) are the major limitation of our study. However, the prospective enrollment of healthy infants without intercurrent respiratory infections is a strength of our paper, which will provide the basis for future physiology study of diaphragm function assessed with a noninvasive procedure. The most important application of electrical impedance tomography (EIT) is as a noninvasive type of medical imaging with ultrasound; in the future, it could be of great importance to study not only the diaphragm, but also the lung gas exchange.

## 5. Conclusions

In conclusion, we provided new data about normal thickening function in healthy children to be used for future study in physiologic and pathologic pediatric studies. This data can be useful to provide reference standards to guide future diagnostic and/or interventional studies involving infants with acute or chronic respiratory conditions.

## Figures and Tables

**Table 1:** Measurements collected during different ultrasound assessments.

	First Echography	Second Echography	Third Echography	Fourth Echography	Fifth Echography
	(N = 22)	(N = 6)	(N = 25)	(N = 24)	(N = 8)
**Weight (Kg)**					
*median (IQR)*	3.23 (2.88–3.41)	3.35 (3.03–3.70)	3.89 (35.60–4.38)	5.50 (5.04–5.92) §	7.44 (6.79–8.11)
*mean (SD)*	3.17 (0.47)	3.38 (0.52)	4 (0.63)	5.52 (0.79) §	7.51 (0.91)
**Chest circumference (cm)**					
*median (IQR)*	33.30 (31.50–34.72)	34.90 (31.12–35.70)	35.50 (34.45–37.05)	39.80 (39.00–41.10) §	45.25 (42.30–46.67)
*mean (SD)*	33.07 (1.63)	33.78 (2.67)	35.80 (2.37)	40.00 (2.33) §	45.14 (3.42)
**Diaphragm thickness in inspiration (mm)**					
*median (IQR)*	27 (20.00–31.75)	25 (23.00–29.25)	30.00 (25.00–33.00)	29.00 (23.50–34.00)	26.00 (22.50–32.50)
*mean (SD)*	27.23 (7.07)	26.17 (7.11)	29.32 (6.52)	28.79 (5.94)	26.38 (5.78)
**Diaphragm thickness in expiration (mm)**					
*median (IQR)*	15 (14–17)	15.50 (12.75–16.25)	16 (13.50–20.00)	19.50 (15.25–22.75)	18 (14.25–18.75)
*mean (SD)*	16 (4.83)	14.50 (2.88)	16.80 (4.90)	19.58 (4.81)	16.50 (2.88)
**Diaphragm Inspiration-expiration ratio**					
*median (IQR)*	1.68 (1.41–1.94)	1.76 (1.54–2.03)	1.69 (1.46–2.19)	1.47 (1.26–1.70)	1.56 (1.43–1.83)
*mean (SD)*	1.80 (0.65)	1.81 (0.35)	1.82 (0.47)	1.50 (0.30)	1.60 (0.20)
Diaphragm thickening fraction *					
*median (IQR)*	0.68 (0.41–0.94)	0.76 (0.54–1.03)	0.69 (0.46–1.19)	0.47 (0.26–0.70)	0.56 (0.43–0.83)
*mean (SD)*	0.80 (0.65)	0.81 (0.35)	0.82 (0.47)	0.50 (0.30)	0.60 (0.20)
Diaphragm thickening fraction **					
*median (IQR)*	0.41 (0.29–0.48)	0.43 (0.35–0.50)	0.41 (0.31–0.54)	0.32 (0.21–0.41)	0.36 (0.30–0.45)
*mean (SD)*	0.40 (0.15)	0.43 (0.09)	0.42 (0.14)	0.31 (0.13)	0.36 (0.08)

* (Diaphragm thickness in inspiration—Diaphragm thickness in expiration)/diaphragm thickness in expiration. ** (Diaphragm thickness in inspiration—Diaphragm thickness in expiration)/diaphragm thickness in inspiration. § Done in 23 children

## Data Availability

Available upon reasonable request to the corresponding author.

## References

[1] Dassios T., Vervenioti A., Dimitriou G. (2022). Respiratory muscle function in the newborn: A narrative review. Pediatr. Res..

[2] LoMauro A., Aliverti A. (2016). Physiology masterclass: Extremes of age: Newborn and infancy. Breathe.

[3] Pham T., Telias I., Beitler J.R. (2020). Esophageal Manometry. Respir. Care.

[4] Weber M.D., Lim J.K.B., Glau C., Conlon T., James R., Lee J.H. (2021). A narrative review of diaphragmatic ultrasound in pediatric critical care. Pediatr. Pulmonol..

[5] Ueki J., De Bruin P.F., Pride N.B. (1995). In vivo assessment of diaphragm contraction by ultrasound in normal subjects. Thorax.

[6] Rehan V.K., McCool F.D. (2003). Diaphragm dimensions of the healthy term infant. Acta Paediatr..

[7] May L.A., Epelman M., Navarro O.M. (2022). Ultrasound imaging of diaphragmatic motion. Pediatr. Radiol..

[8] Buonsenso D., Supino M.C., Giglioni E., Battaglia M., Mesturino A., Scateni S., Scialanga B., Reale A., Musolino A.M.C. (2018). Point of care diaphragm ultrasound in infants with bronchiolitis: A prospective study. Pediatr. Pulmonol..

[9] Alonso-Ojembarrena A., Ruiz-González E., Estepa-Pedregosa L., Armenteros-López A.I., Segado-Arenas A., Lubián-López S.P. (2020). Reproducibility and reference values of diaphragmatic shortening fraction for term and premature infants. Pediatr. Pulmonol..

[10] El-Halaby H., Abdel-Hady H., Alsawah G., Abdelrahman A., El-Tahan H. (2016). Sonographic evaluation of diaphragmatic excursion and thickness in healthy infants and children. J. Ultrasound Med..

[11] Buonsenso D., Berti B., Palermo C., Leone D., Ferrantini G., De Sanctis R., Onesimo R., Curatola A., Fanelli L., Forcina N. (2020). Ultrasound assessment of diaphragmatic function in type 1 spinal muscular atrophy. Pediatr. Pulmonol..

[12] Berti B., Buonsenso D., De Rose C., Ferrantini G., De Sanctis R., Forcina N., Mercuri E., Pane M. (2022). Point-of-care lung and diaphragm ultrasound in a patient with spinal muscular atrophy with respiratory distress type 1. J. Ultrasound.

[13] Zambon M., Greco M., Bocchino S., Cabrini L., Beccaria P.F., Zangrillo A. (2017). Assessment of diaphragmatic dysfunction in the critically ill patient with ultrasound: A systematic review. Intensive Care Med..

[14] Pellegrini M., Hedenstierna G., Roneus A., Segelsjö M., Larsson A., Perchiazzi G. (2017). The Diaphragm Acts as a Brake during Expiration to Prevent Lung Collapse. Am. J. Respir. Crit. Care Med..

